# Various gene modification techniques to discover molecular targets for nonhormonal male contraceptives: A review

**DOI:** 10.18502/ijrm.v21i1.12662

**Published:** 2023-02-08

**Authors:** Luluk Yunaini, Dwi Ari Pujianto

**Affiliations:** ^1^Doctoral Program of Biomedical Sciences, Faculty of Medicine, Universitas Indonesia, Jakarta Pusat, Indonesia.; ^2^Department of Medicine Biology, Faculty of Medicine, Universitas Indonesia, Jakarta Pusat, Indonesia.

**Keywords:** Gene modification, Nonhormonal, Male contraceptive.

## Abstract

The identification and characterization of relevant targets are necessary for developing nonhormonal male contraceptives. The molecules must demonstrate that they are necessary for reproduction. As a result, a sophisticated technique is required to identify the molecular targets for nonhormonal male contraceptives. Genetic modification (GM) techniques are one method that can be applied. This technique has been widely used to study gene function that effected male fertility and has resulted in the discovery of numerous nonhormonal male contraceptive target molecules. We examined GM techniques and approaches used to investigate genes involved in male fertility as potential targets for nonhormonal contraceptives. The discovery of nonhormonal contraceptive candidate molecules was increased by using GM techniques, especially the Clustered Regularly Interspaced Short Palindromic Repeats/Cas9 method. The discovery of candidate nonhormonal contraceptive molecules can be a wide-open research for the development of nonhormonal male contraceptives. Therefore, we are believing that one day nonhormonal male contraceptives will be released.

## 1. Introduction 

Until now, women dominated contraceptive methods, and men were under-represented (1, 2). Despite varying contraceptive methods, unplanned pregnancy rates remained high (3-5). Male contraceptives include condoms and vasectomy. Contrary to popular belief, vasectomy is irreversible and less reliable than female contraception (4). Hormonal male contraceptives work by interrupting the negative feedback loop of hypothalamus-pituitary-gonad axis. This method of contraception should be revisited due to several problems and negative effects. Nonhormonal male contraceptives have been created to avoid male hormonal contraceptive side effects (6). One of the candidates for nonhormonal male contraceptives is the epididymal molecule which is suspected to play a role in sperm maturation (7-10). The development of nonhormonal male contraceptives by targeting proteins or genes involved in male fertility is promising for controlling male fertility. A consistent strategy for investigating gene function is necessary to identify candidate genes for use as biomarkers or contraceptive targets. The best way to determine gene function is by genetic modification (GM) techniques in animal model. Common GM approaches include transgenic, knockdown, and knockout/knock-in/gene-trapped (11, 12).

More than 400 genes that play a role in male fertility have been analyzed using animal models (11). This number is less because thousands of genes play a role in the regulation of the complex process in male fertility. GM techniques are developing rapidly. This was followed by an increasingly fast and easy way to make animal models which resulted in more findings of genes that play a role in male fertility as candidates nonhormonal male contraceptives. Although there are many candidate molecular targets for nonhormonal male contraceptives, the development and progress of nonhormonal male contraceptives are slow. The scope of the problem in this article is GM techniques for producing animal models, various genes that play a role in male fertility as nonhormonal male contraceptive candidates, and the development of nonhormonal male contraceptives with reported molecular targets.

## 2. Material and Methods

The research for relevant articles to this review was acquired from search engines particularly Google Scholar, PubMed, Elsevier, and Scopus distributed between January 2003-December 2021. The keywords used were contraceptive, nonhormonal, GM techniques, animal model, and molecule candidate of male contraceptive. The articles were observed and specified for the current review. This study concentrates on discovering molecule candidates of nonhormonal male contraception from various GM techniques. We categorize 13 articles about the generation of making animal models for infertility by using transgenic, knockout, or Clustered Regularly Interspaced Short Palindromic Repeats/Cas9 (CRISPR/Cas9) techniques. A total of 27 articles in which researchers examined genetically modified to produce sterile or decreased fertility in males were found. Genes that affect infertility in males but do not affect hormones were used as candidates for nonhormonal male contraceptives. Furthermore, a search for candidate nonhormonal male contraceptive molecules was conducted and 26 articles were collected.

## 3. Results

### Animal model using GM 

More than thousand genes are involved in spermatogenesis. As spermatogenesis is complex and there are multiple causes of aberrant spermatogenesis, using experimental animal models with genetic alteration techniques is particularly advantageous. Mice are the most often used experimental animals because they have a short reproductive cycle, genetics similar to humans, and mouse embryos are easily genetically modified (12). Using GM techniques to create experimental animals allows genes from one individual to be transferred to another or between species.

#### Transgenic

**Figure 1:**
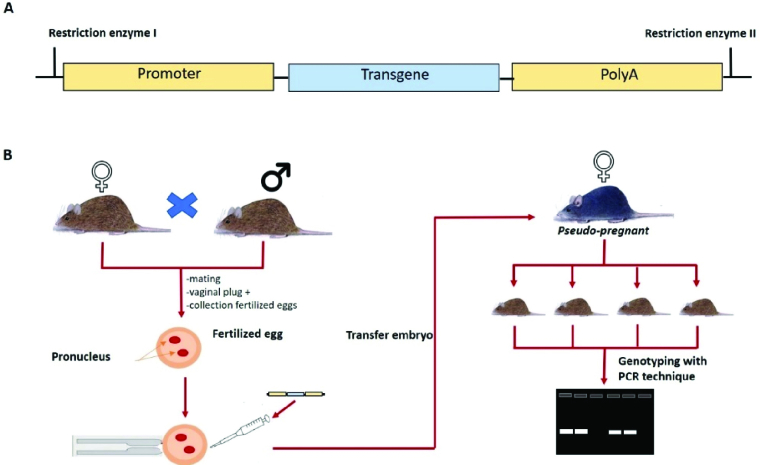
Transgenic mouse production. A) The transgene construct contain promoter expressed transgenes intronic and pattern denylation sequences. B) Illustrates the procedure for producing transgenic mice. Collection fertilized egg, injected by target gen and implanted into the uterus of pseudopregnant female mice under direct visualization. The genotypes of transgenic mice were determined using a PCR technique.

Transgenics are genetic alterations generated through recombinant DNA insertion that can be passed down from generation to generation. Endogenous or exogenous genes or DNA fragments are introduced to boost expression (11). Initially, transgenic individuals were generated through nuclear transfer and microinjection into the pronucleus. Microinjection has persisted because it is more stable than nuclear transfer (12). Figure 1 showed the process of creating transgenic animals.

The microinjection transgenic technique has a number of advantages and disadvantages. The advantages are that almost all cells from these transgenic offspring contain transgenes, production is relatively quick, and the effects of gene overexpression can be analyzed and studied to determine the gene's utility. While disadvantages are integrated randomly in a small percentage of cases, there is a risk of DNA entering critical loci, resulting in genetic mutations, nonphysiological phenotypic effects. Transgenes can enter gene loci silencing (13, 14).

#### Knockout 

**Figure 2:**
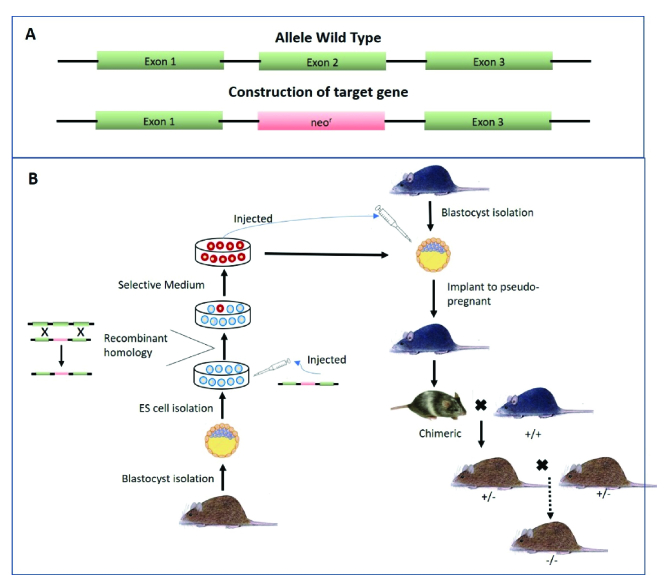
The method of gene knockout utilizes embryonic stem (ES) cells. A) Construction of allele wild type and gene target. B) ESCs was isolated and cultured from the inner cell mass of wild-type mice blastocysts. Selected ES cells were injected into blastocysts from the other strains and implanted into pseudopregnant female mice. Chimeric progeny will be produced. To generate heterozygous knockout offspring, chimeric offspring were mated with wild-type mice. Heterozygous offspring can be mated to generate homozygous knockout mice.

The term “knockout" refers to animals whose target genes have been deleted by deleting critical exons to prevent transcription. One strategy for deactivating a gene is to delete its critical exon and replace it with an antibiotic-resistant gene, such as the neomycin gene (*neo ${}^{r}$ *). After electroporation of the constructed gene into embryonic stem cells (ESCs), the cells are grown in a medium containing the antibiotic neomycin. As a result, the transgenic ESCs will survive in a neomycin-treated medium. Additionally, ESCs containing recombinant DNA will be injected into the blastocyst to generate chimeras. Then, chimeric organisms are mated with wild-type organisms to generate heterozygous offspring. This heterozygous offspring will be crossed with another heterozygous offspring to generate homozygous mutant/knockout offspring (12). Figure 2 depicts a production knockout animal model.

Despite knockout widespread use, this technique is still time-consuming and expensive than transgenic techniques. Knockout can result lethal embryonic or neonatal death caused by target gene is expressed at early embryonic or many tissues that are important for support in neonatal. So they cannot be evaluated postnatally or in adulthood. Another disadvantage of standard knockout is the difficulty distinguishing direct effects on cells or tissues from secondary effects on other organs caused by indirect actions. As a result, it is critical to keep in mind that the observed phenotype may result from both direct and indirect actions of gene products (12, 15). The solution to these problems is to develop knockout techniques such as conditional knockout and knockout inducer. Conditional knockout occurs in specific tissues and organs using the cloning vector pAW8-yEGFP (Cre)-locus of X over (lox) system. The Cre-Lox system comprises 2 components: the Cre recombinase and the recognition site loxP (16).

**Figure 3:**
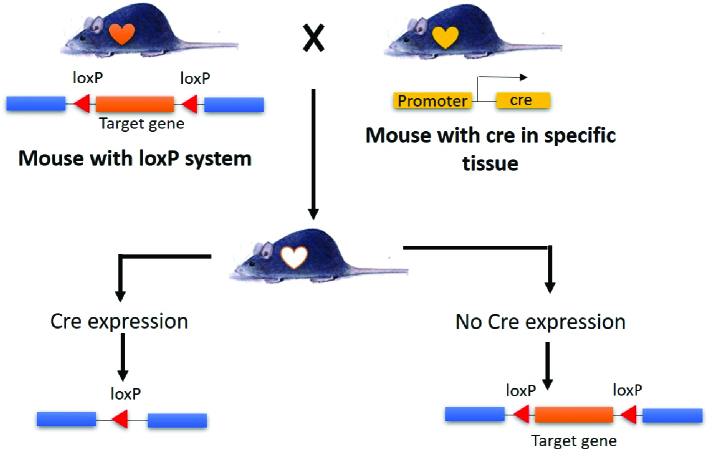
The Cre-loxP system is used to generate conditional knockout experimental animals. Animals with lox in the target gene and animals with Cre expressed exclusively in certain organs/tissues. If Cre is expressed, the offspring will be knockout; if Cre is not expressed, the offspring will be wild type.

2 mutant animal strains must be used to generate mutants using the Cre-loxP system. The Cre strain was expressed in a specific tissue or organ in the first animal. The second animal is a strain of animal that has a loxP recognition site flanking the target gene that will be excised/cut in a unidirectional orientation. Additionally, the offspring of these 2 animal strains were mated to ensure that target genes were knocked out only in specific tissues/organs. This occurs because Cre is expressed in a limited number of tissues/organs. The Cre protein recognizes the loxP recognition area between the target gene, resulting in the target gene's excision and truncation (17). Procedure conditional knockout can be seen in figure 3.

The advantages of conditional knockout are flexibility of the Cre-loxP system, can assess the role of target genes at various developmental stages, and enables analysis of the target genes role at a variety of sites. The disadvantage of this method is that it is difficult to identify a promoter that will specifically direct Cre expression to the desired tissue (16, 17).

Knockout induction (Inducible Cre-loxP system) technique enables precise targeting of Cre activation at specific times and in specific cells. Cell-specific regulatory elements (promoters and enhancers) regulate the inducible Cre system. Tamoxifen (tam) and tetracycline (tet) are 2 frequently used exogenous inducers. The tam-induced Cre-loxP system operates on the principle that when Tam is not present, Cre-Estrogen Receptor (CreER) interacts with heat shock protein 90 and is localized in the cytoplasm. If present in the cytoplasm, Tam inhibits the interaction between heat shock protein 90 and CreER. CreER is translocated to the nucleus. CreER recognizes the loxP site in the nucleus and inactivates target gene in specific tissue. Inducible cre-loxP system using tet is triggered by Dox. This induction is available in 2 modes: Cre (Dox) Tet-on and Cre (Dox) Tet-off. In the Tet-on system, the presence of Dox will induce Cre expression and make deletion of the target gene, whereas in the Tet-off system the presence of Dox inhibit Cre expression and make the target gene expression (17).

The advantage of induced knockout is that it enables precise control of the target gene's expression time, allowing us to avoid the presence of lethal embryos in genes involved in embryology, such as those involved in the cardiovascular system (18). Additionally, this technique can investigate lineage cell tracing. The inducer can substantially affect the phenotype, which is a limitation of the inducible Cre model. For instance, regardless of the target gene's deletion, an induced Cre model with tam would significantly affect estrogen-responsive organs such as bone, uterus, breast, and liver (19).

#### Clustered regularly interspaced short palindromic repeats (CRISPR) 

**Figure 4:**
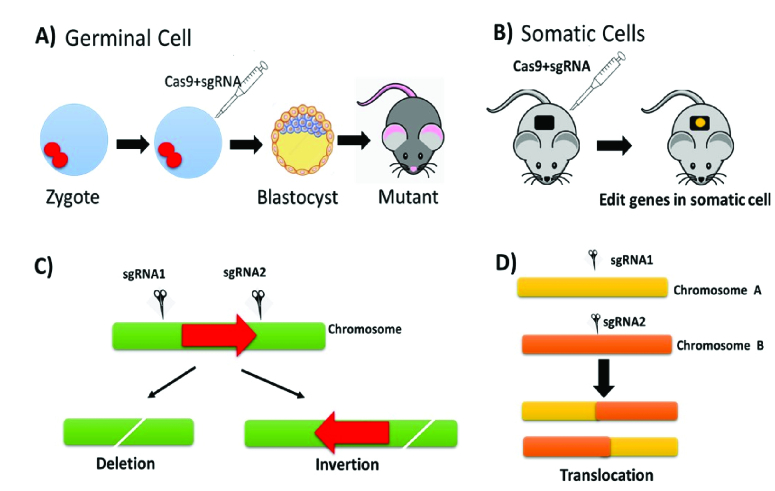
Making experimental animals with CRISPR/Cas9: A strategy A) Microinjection of CRISPR/Cas9 into the pronucleus of fertilized egg cells. B) The use of CRISPR/Cas-9 to somatic cells. C) Using CRISPR/Cas-9 in conjunction with 2 adjacent sgRNAs resulted in the deletion or inversion constructs, which resulted in the gene ceasing to express. D) When 2 sgRNAs are used on distinct chromosomes, a translocation can occur.

CRISPR is a type of DNA segment found in prokaryotes (bacteria and archaea) consisting of sequences or sequences of nucleotides with short repetitions. The *CRISPR* gene functions as an immune system in these prokaryotes, protecting them from infection, conjugation, and transformation caused by foreign genetic material (14). CRISPR-Cas9 can be used to edit genes depending on the system's capability. In contrast to transgenic techniques, which perform genetic engineering on a genome randomly, the CRISPR-Cas9 system performs targeted and precise genetic engineering (Figure 4) (20).

The primary benefit of this technology is its ability to automate complex gene targeting vector designs and ESCs manipulation, thereby reducing modeling time and costs. Additionally, CRISPR/Cas9 is not restricted to a rodent's particular strain or genetic background, as ESCs is not required (21). CRISPR-based gene editing makes knockout easier.

One disadvantage of the CRISPR/Cas9 system is that it is inefficient at inserting large DNA sequences (22). To overcome this limitation, it was reported in 2017 that the addition of a CRISPR single stranded DNA (ssDNA) insert (Easi-CRISPR) could be used. These donor sequences can range in length from 500 nt to 2 kb and have been shown to provide highly efficient targeted insertion (23).

The advantage of Easi-CRISPR is that long donor ssDNA sequences do not randomly integrate into the genome, as is the case with short donor dsDNA sequences. Additionally, this donor serves as a superior template for homology repair compared to conventional CRISPR sequences. While Easi-CRISPR appears to be a significant advancement over basic CRISPR technology, ensuring the quality and precision of extremely long ssDNA sequences. The resulting RNA will be reverse-transcribed using reverse transcriptase to generate this sequence. Unlike DNA-dependent polymerases, this enzyme is prone to errors and cannot correct them. This results in sequences that may contain mutations (23).

### Male infertility genes as a nonhormonal contraceptive candidate

The testes and epididymis are 2 organs that are involved in the process of male infertility. The testes are involved in spermatogenesis, while the epididymis is involved in sperm maturation. As a result, numerous studies have examined male infertility at the genomic, transcriptomic, and proteomic levels in these 2 organs. Along with organ analysis, molecular analysis at the cellular level, specifically spermatozoa, was performed. Obtaining a contraceptive target molecule is one of the objectives of the molecular study of male infertility.

The number of genes and proteins thought to play a role in male fertility must be established by understanding their role and function in fertility. GM techniques, particularly knockout, are one method for analyzing the function and role of genes. Fertility analysis must be performed in vivo due to the complexity of the reproductive process. As a result, it is essential to analyze the function and role of male fertility genes. In table I, we list the genes involved in fertility that have been characterized using knockout animal models.

**Table 1:** Gene list which has a role in male fertility using GM techniques


**Gene name**	**Phenotype**	**Reference**
*Defb41*	Changes in sperm motility and decreased bonding with the egg by in vitro study	24
*Defb23/26, Defb23/26/42*	Subfertility, decreased motility, premature capacitation	22
*Defb1/2/9/10/11/13/15/35/ 50*	Decreased sperm motility, increased sperm fragility, premature capacitation, decreased bonding with the egg	25
*Brca2*	Spermatocytes stop early in prophase I	26
*Crisp2/Crisp4*	Inflammation of the epididymis resulting in decreased sperm viability and decreased fertility rates	27
*Catsper and KSper*	Infertility without apparent systemic effects	28
*Ercc1*	Increased DNA damage in testes, increased apoptosis in germ cells	29
*Eif4g3*	Stopped at the stage of meiosis, spermatocytes fail to exit prophase through the G2/MI transition	30
*Eppin*	Reduction of sperm motility	31
*Gpx4*	Sperm count decreased significantly, infertility in males, reduced sperm motility, swollen mitochondrial membrane	32
*Fkbp6*	Stopping at the pachytene stage and increasing apoptosis in germ cells	33
*Fimp*	Completely infertility or severely subfertile	34
*H1fnt*	Oligoasthenoteratospermia, DNA fragmentation, spermatids contain a lot of residual cytoplasms	35
*Hipk4*	Infertile	36
*Izumo1*	Infertile	37
*Lmtk 2*	Stop at spermatid stage, azoospermia	38
*M2y2 (Ybx2)*	Abnormal and multinuclear sperm morphology in spermatids	39
*Nanos3*	Total loss of spermatogonia	40
*Paf-ah1b1 (Lis1)*	Acrosome damage and tail formation	41
*Rec8*	Abnormal sister chromosome synapses, azoospermia	42
*Slo3*	Male infertility	43
*Spink2*	Oligoasthenoteratospermia to azoospermia	44
*Sof1, Spaca6*	Completely infertile or severely subfertile	45
*Tekt2*	Sperm motility decreased significantly	46
*Tmem95*	Completely infertile or severely subfertile	45
*Tssk1/Tssk2*	Infertility	47
*Defb*: Beta defensin, *Brca2*: Breast cancer 2, *Crisp*: Cysteine-RIch secretory protein, *Catsper/KSper*: Cation channel, sperm associated 1, *Ercc1*: Excision repair cross-complementing rodent repair deficiency, complementation group 1, *Eif4g3*: Eukaryotic translation initiation factor 4 gamma, 3, *Eppin*: Epididymal peptidase inhibitor, *Gpx4*: Glutathione peroxidase 4, *Fkbp6*: FK506 binding protein 6, *Fimp*: Fertilization influencing membrane protein, *H1fnt*: H1.7 linker histone, *Hipk4*: Homeodomain interacting protein kinase 4, *Izumo1*: Izumo sperm-egg fusion 1, *Lmtk 2*: Lemur tyrosine kinase 2, *M2y2/Ybx2*:Y box protein 2, *Nanos3*: nanos C2HC-type zinc finger 3, *Paf-ah1b1*: Platelet-activating factor acetyl hydrolase, isoform 1b, subunit 1, *Rec8*: REC8 meiotic recombination protein, *Slo3/Kcnu1*: Kalium channel, subfamily U, member 1, *Spink2*: Serine peptidase inhibitor, Kazal, *Sof1*: Sperm-oocyte fusion required 1, *Spaca6*: Sperm acrosome associated 6, *Tekt2*: Tektin-t, *Tmem95*: Transmembrane protein 95, *Tssk*: Testis specific ser/thr kinase		

Spermatozoa become fertile during their transit through the epididymis. Microarray analysis identified over 17,000 genes expressed in the epididymis, but only a few are expressed in the epididymis to a limited extent. To investigate the function of highly expressed genes in the epididymis in vivo, experimental animals in the form of mice were created that were deficient in 9 genes found to be highly expressed in the head and corpus epididymis (Pate1, Pate2, Pate3, Clpsl2, Epp13, Rnase13, Gm1110, Glb1l2, and Glb1l3). The CRISPR/Cas9 system was used to generate knockout mice. The epididymis histology and sperm morphology of all knockout lines were identical to those of control males. Females of wild-type mated with knockout males produced the same number of offspring as control males. Thus, 9 genes that were abundantly expressed in the head and corpus epididymis were discovered to be secreted for sperm function and male fertility. The generation of knockout mice using CRISPR/Cas9 accelerates screening genes expressed in the epididymis for potential reproductive functions (48).

### Development of nonhormonal male contraceptives

Nonhormonal male contraceptives have progressed to the preclinical stage by targeting sperm production or function proteins. The number of target molecules with significant potential as nonhormonal male contraceptives increases year after year. Controlling sperm production or function takes a variety of forms, and each form must be evaluated to ensure that it is both safe and effective as a contraceptive. According to animal models, the target may be effective if specificity is increased to limit off-target effects.

The first candidate molecule for nonhormonal male contraceptives is retinoic acid. It was discovered that male sterility could be caused by vitamin A deficiency (49). Table II showed details of the development of nonhormonal male contraception.

**Table 2:** Development of nonhormonal male contraceptive


**No**	<**Molecule target/mechanism of action**		**Small molecule of contraception candidate**			**Note**
	**Name**	**Function**	**Name**	**Role**	**Response**	
		WIN18.446	Analog in the Retinoic acid (RA) pathway	Efficacious and well tolerated in inhibiting spermatogenesis (50)	It was halted because consuming alcohol while taking this drug results in a severe disulfiram reaction (51)
**1.**	Retinoic acid
		receptor (RAR)	Inhibiting
		spermatogenesis	
		(50)		BMS-189453	RAR antagonist	Reversible and inhibition of spermatogenesis in the mouse model (52)	Screening for a strong specific antagonist RAR using in silico method to inhibiting spermatogenesis (6)
**2.**	Bromodomain, testis specific (BRDT)	Plays an important role in chromatin remodeling during spermatogenesis (53, 54)		JQ1	BRDT inhibitor	Reversible contraceptive effect in male rats (55) but can Binds to other BRD proteins that do not target molecules (56)	It is necessary to develop optimized BRDT inhibitors. The potent and selective BET inhibitor candidate for BD2 was revealed by in silico analysis (6)
**3.**	Kalium channel, subfamily U, member 1 (Kcnu1/Slo3)	Controls calcium influx through CatSper. SLO3 genetic deletion causes infertility in male mice (57)	RU1968	Steroid inhibitor	CatSper progesterone-mediated motility dysfunction through Inhibition of hSLO3 (58)	It is unclear whether this approach would be more suitable for female use because of the impact on progesterone function in the fallopian tubes (58)
**4.**	Epididymal peptidase inhibitor (EPPIN)	Sperm motility (31)	EP055	EPPIN inhibitor	Significant reduction in sperm motility reduce through decreasing sperm internal pH and rapid calcium levels (59, 60)	Preclinical study
		CDB-4022	Mitogen-activated protein kinase (MAPK) pathway activation in Sertoli-germ cell junction	Inhibits the mature sperm (61)	Reversible decrease in sperm production with no apparent side effects (61)
**5.**	Sertoli cell	Sperm maturation
			(61-63)	Indazole carboxylic acid derivatives such as Gamendazole, H2-gamendazole, and Adjudin	Disorder of Sertoli cell adhesive junction protein	In rats treated orally with fertility was inhibited by H2-gamendazole in rat (62) The effect is reversible at low-drug doses, and irreversible at higher doses. Adjudin was conjugated to the recombinant FSH binding fragment to specifically target the testicular germ cell-Sertoli germ cell junction (63)	
			**6.**	Testis-specific serine/threonine kinases (TSSK)	Spermatogenesis and sperm function (53)	GSK2163632 A	TSSK2 inhibitor	Loss of fertility in mice (53)	It is necessary to identify selective molecules in inhibiting TSSK2 because this compound can inhibit other kinases than TSSK2. Inconsistent side effects was leaded by the nonselective action of this molecule (53)
			**7.**	CatSper	Capacitation, hyperactivation of motility, and the acrosome reaction (54)	Nifedipine	Calcium channel blocker (54)	Epididymal sperm counts, motility, and fertility of male BALB/c mice significantly decreased (54)	
				**Name**	**Function**	**Name**	**Role**	**Response**	
			**8.**	Vasopression receptor	Sperm count and motility (64)	Deamino [Cys 1, D-ArgS] vasopressin (dDAVP)	Vasopressin receptor (AVPR2) agonist (64)	Decreased intracellular pH, PKA substrates, sperm motility, and increased Ca2+ concentration (64)	
**9.**	Actin-related protein 2/3 (Arp2/3)	Sperm motility and capacitation (65)	CK-636	Arp2/3 complex antagonist	This molecule induced hyper-activated motility and acrosomal reaction, besides that spermatozoa were inhibited by intracellular calcium and tyrosine phosphorylation levels (65)	Reduced fertilization and embryo development in the highest concentration of CK-636, but after treatment, fertilization significantly increased, whereas embryonic development significantly decreased (65)
**10.**	Na, K-ATPase (NKA)	Sperm motility (66)	Ouabain	Inhibitor of NKA activity (66)	Reduction in sperm motility (66)	
**11.**	Ca-ATPase of the plasma membrane (PMCA)	Sperm motility (66)	Amiloride	Inhibitor of PMCA activity (66)	Reduction in sperm motility (66)	
**12.**	Na(+)/Ca (2) (+)-exchanger (NCX)	Sperm motility (66)	Eosin	Inhibitor of NCX activity (66)	Reduction in sperm motility (66)	
**13.**	Na(+)/H(+)-exchanger (NHE)	Sperm motility (66)	KB-R7943	Inhibitor of NHE activity (66)	Reduction in sperm motility (66)	
**14.**	Dynein-ATPase activity	Sperm motility (66)	Acidic pH and micromolar concentrations of Ca2+	Inhibitor of Dynein-ATPase activity (66)	Inhibition of sperm motility through higher cytosolic H(+) and Ca (2) (+) (66)	
**15.**	α1-adrenoceptor	Contribute to ejaculation (67)	Tamsulosin, prazosin	α1-adrenoceptor antagonist (67)	Decreased in male fertility, mating uninhibited and reversible libido (67)	It causes an increase in preimplantation losses and a significant decrease in ejaculatory competence (67)
**16.**	Serotonin-norepinephrine reuptake	Control sperm transit time (68)	Sibutramine	Non-selective serotonin-norepinephrine reuptake inhibitor (68)	Reduced sperm quality (68)	
**17.**	Glyceraldehyde 3-phosphate dehydrogenase, spermatogenic (GAPDHS)	Spermatogenesis (69)	(S)-α-chlorohydrin (SACH)	GAPDHS inhibitor	Disturbed spermatogenesis through blocking the cAMP/PKA pathway in sperm (69)	
**18.**	Adenylyl cyclase-10 (ADCY10)	The primary enzyme responsible for the production of cAMP in sperm (70)	CE (2HE) KH7 DIDS ASI-8 Bithionol LRE1	S-Allylcysteine (sAC) inhibitors	Decreased male fertility through reduced sperm motility and capacitation (70)	The correlation between dominant absorptive hypercalciuria and ADCY10 has not slowed the development and optimization of ADCY inhibitors as nonhormonal contraceptives (70)
WIN18.446: N,N'-1,8-Octanediylbis (2,2-dichloroacetamide), BMS-189453: (4-[(1E)-2-(5,6-Dihydro-5,5-dimethyl-8-phenyl-2-naphthalenyl)ethenyl]-benzoic acid, JQ1: ((S)-tert-butyl 2-(4-(4-chlorophenyl)-2,3,9-trimethyl-6H-thieno[3,2-f,1,2,4]triazolo[4,3-a,1,4]diazepin-6-yl)acetate), RU1968: ((1R,3aR,3bR,9bR,11aR)-1-[(1R)-1- { [2-(dimethylamino)ethyl]amino } ethyl]-11a-methyl-1H,2H,3H,3aH,3bH,4H,5H,9bH,10H,11H,11aH-cyclopenta[a]phenanthren-7-ol)						

Research on genetic variation of nonhormonal contraceptive target molecules with male fertility has been carried out. Several genes showed associations between gene variations of target molecule and male infertility. ADCY10 is the only soluble adenylate cyclase in the ADCY1-10 family of proteins. Recent whole-exome sequencing of the 2 infertile men revealed that their asthenozoospermia condition, which affects progressive sperm motility, was caused by a homozygous variant upstream of the ADCY10 nucleotide- binding site, which results in premature sperm termination (71). These individuals live normally but are infertile and have a risk of developing calcium oxalate kidney stones.

The testis-specific serine/threonine kinases (TSSK) family consists of 5 members: TSSK1, TSSK2, TSSK3, TSSK4 (also referred to as TSSK5), and TSSK6. Several of its members are critical for spermatogenesis and sperm function. The production of a stable and enzymatically active recombinant human TSSK2 protein represents a significant step forward to eradicate TSSK in humans (72). Furthermore, when men with azoospermia or severe oligozoospermia were compared to fertile controls, single nucleotide polymorphisms in the *TSSK2* gene were associated with idiopathic male infertility (73). TSSK2 essay screening revealed a potent TSSK2 inhibitor. This demonstrates the great potential for targeting TSSK with small molecule inhibitors (53, 74).

## 4. Discussion 

Proteomic analyses have been performed on the male reproductive system and the distribution of gene expression in various tissues. Numerous genes were found to be overexpressed in the male reproductive system due to this study's findings. Numerous reports have implicated genes in spermatogenesis and sperm maturation (2). These findings pave the way for the development of candidate target molecules for nonhormonal male contraceptives. For nonhormonal contraception, at least 3 molecular targets are involved in spermatogenesis, sperm maturation, and sperm penetration into oocytes. As a result, numerous analyses of proteins involved in these 3 processes or molecules with high-expression levels in spermatozoa, testes, and epididymis have been conducted.

The high expression in target organs or cells for nonhormonal male contraceptives is frequently accompanied by an ambiguous function and role in male reproduction. Thus, it is critical to employ GM techniques in experimental animals. When developing experimental animals, it is necessary to consider specific genetic mechanisms that disrupt gene function, alter gene activity, or increase gene copy number. If a phenotype is caused by a complete loss of function (zero mutation), the gene can be silenced using gene targeting (gene knockout). Gene knockout can model homozygous recessive traits (inactivation of both copies of the gene by mutation) and haploinsufficient traits (inactivation of one copy of the gene). Transgenic techniques can be used when the phenotype is caused by an increase or new activity of a gene product or an increase in the number of gene copies. If a mutation results in a partial loss of function, it is possible to introduce specific harmful mutations into endogenous genes using gene targeting (knock-in). Additionally, it should be noted that phenotypic changes are not always caused by a loss of gene function in its entirety (null mutation). However, because the null mutation provides the most information about the gene's normal function, it can be used to deduce the molecular mechanism underlying the phenotype (75).

The considerations outlined above can guide the selection of experimental animals using the GM technique. The most recent and most advanced GM technique is CRISPR/Cas-9 for gene editing. This technique can precisely knockout or knock in the target gene. This technique requires less time than other methods, which enables rapid analysis of the function and role of a gene.

Male fertility research is generally associated with genes expressed during the early embryonic and puberty stages. Genes expressed in early embryos are involved in gonad formation and the development of the reproductive tract. Genes are involved in the process of spermatogenesis and sperm maturation during puberty. Numerous genes expressed early in the embryo are also expressed in specific tissues/organs during puberty. The use of gene editing, specifically CRISPR/Cas-9, is optimal for the study of male fertility because the time required to construct the target gene vector is shorter, more precise, and can be combined with the Cre-loxp system (21).

Furthermore, numerous related genes, such as the Crisp and Defb relatives, play a significant role in male infertility. Previous studies have shown that knocking out just one member of a relative gene produces a phenotype identical to the wild type. This is because related genes perform similar functions, and it is suspected that if only one member is deleted, the cell compensates. As a result, it is necessary to eliminate more than one member (22, 27). Thus, combining CRISPR/Cas-9 with the Cre-loxP system accelerates the process and ensures that knockout occurs only in the target tissue or organ. Candidate molecular targets for nonhormonal male contraception will be more precise, specific, and effective using this technique.

Men, according to surveys, want highly effective and irreversible contraception. However, male contraceptives, particularly nonhormonal contraceptives, have developed at a glacial pace until now. The development of GM techniques for analyzing the function and role of genes in the male reproductive system-aided significantly in developing male contraceptive target molecules. Until now, nonhormonal male contraception has been developed in the preclinical stage using experimental animals. The results of experiments on animals frequently differ from one another. This is partly due to the background variations of the experimental animals used, particularly those using ES cells, which have a different background than the blastocyst used, resulting in tillers with a wide range of background variations. These contrasting backgrounds can be minimized by crossing up to several offspring and using the resulting offspring.

It should be noted that when using an experimental animal technique without ESCs, different strains can also produce distinct mutation effects. As a result, it is necessary to analyze the gene in relation to the animal's background. Utilizing the CRISPR/Cas-9 technique makes it possible to minimize background variation in the animals used, as the CRISPR/Cas-9 technique does not require ES cells. As a result, it is necessary to analyze the gene in relation to the animal's background.

The analysis of gene function and its role in the male reproductive system cannot be applied directly to humans. This is because some genes' function and biological function in mice/experimental animals may differ from those in other species. As a result, preclinical testing is frequently conducted on higher-level animals before human testing. SNP analysis and gene expression analysis of candidate contraceptive targets in infertile men can also be used to ascertain the gene-gene association with male infertility. Human ortholog gene analysis is typically performed on gene family members to determine whether the target gene in experimental animals shares a high degree of homology with the human gene.

Once the target molecule is obtained, screening is required to determine which drug molecule can be used as an inhibitor or anti-molecule target antibody. Frequently, when a target molecule has multiple isoforms, it is necessary to analyze the specific bonds and high efficacy of the isoforms. Additionally, if the target molecule is expressed in multiple organs, it will be considered. The first step should be to determine the presence or absence of target molecule isoforms expressed specifically in male reproductive organs such as the testes or epididymis. Moreover, small molecules are synthesized that inhibit the activity of the specific target molecule. The candidate drug molecule must exhibit ideal contraceptive properties, apart from inhibiting the target molecule.

## 5. Conclusion

A good contraceptive must meet a number of criteria, including being safe, reliable, effective, affordable, easy to use, without serious side effects, readily available, and reversible. It will take a long time to develop the ideal nonhormonal male contraceptive. However, with the rapid advancement of research in the field of reproduction, particularly male reproduction, and the continued innovation of GM techniques especially with CRISPR-Cas9 discovery as an editing gene. The rapid advancement of GM techniques may enable the identification of more precise and specific molecular targets for nonhormonal male contraceptives. So, we are optimistic that one day nonhormonal male contraceptives will be developed and it will be effective and beneficial to the wider community.

##  Conflict of Interest 

The authors declare that there is no conflict of interest.

## References

[B1] Naz RK, Rowan Sh (2009). Female contraception: Present and future perspectives. Curr Womens Health Rev.

[B2] Arifuzzaman S, Rahman MS, Pang M-G (2019). Research update and opportunity of non-hormonal male contraception : Histone demethylase KDM5B-based targeting. Pharmacol Res.

[B3] Finer LB, Zolna MR (2016). Declines in unintended pregnancy in the United States, 2008-2011. N Engl J Med.

[B4] Sundaram A, Vaughan B, Kost K, Bankole A, Finer L, Singh S, et al (2017). Contraceptive failure in the United States : Estimates from the 2006-2010. Perspect Sex Reprod Health.

[B5] Eisenstein M

[B6] Long JE, Lee MS, Blithe DL

[B7] Pujianto DA, Loanda E, Sari P, Midoen YH, Soeharso P (2013). Sperm-associated antigen 11A is expressed exclusively in the principal cells of the mouse caput epididymis in an androgen-dependent manner. Reprod Biol Endocrinol.

[B8] Pujianto AD, Muliawati D, Dara M, Parisudha A, Hardiyanto L (2020). Mouse defensin beta 20 (Defb20) is expressed specifically in the caput region of the epididymis and regulated by androgen and testicular factors. Reprod Biol.

[B9] Browne JA, Leir SH, Yin S, Harris A (2019). Transcriptional networks in the human epididymis. Andrology.

[B10] Pujianto DA, Permatasari S (2021). Mouse CD52 is predominantly expressed in the cauda epididymis, regulated by androgen and lumicrine factors. J Hum Reprod Sci.

[B11] Jamsai D, O’Bryan MK (2011). Mouse models in male fertility research. Asian J Androl.

[B12] Tamowski S, Aston KI, Carrell DT (2010). The use of transgenic mouse models in the study of male infertility. Syst Biol Reprod Med.

[B13] Conrad DF, Pinto D, Redon R, Feuk L, Gokcumen O, Zhang Y, et al (2010). Europe PMC funders group origins and functional impact of copy number variation in the human genome. Nature.

[B14] Lampreht Tratar U, Horvat S, Cemazar M (2018). Transgenic mouse models in cancer research. Front Oncol.

[B15] Takehashi M, Kanatsu-Shinohara M, Shinohara T (2010). Generation of genetically modified animals using spermatogonial stem cells. Dev Growth Differ.

[B16] Horii T, Morita S, Kimura M, Terawaki N, Shibutani M, Hatada I (2017). Efficient generation of conditional knockout mice via sequential introduction of lox sites. Sci Rep.

[B17] Kim H, Kim M, Im S-K, Fang S (2018). Mouse Cre-LoxP system : General principles to determine tissue-specific roles of target genes. Lab Anim Res.

[B18] Andersson KB, Winer LH, Mork HK, Molkentin JD, Jaisser F (2010). Tamoxifen administration routes and dosage for inducible Cre-mediated gene disruption in mouse hearts. Transgenic Res.

[B19] Ye R, Wang QA, Tao C, Vishvanath L, Shao M, Mcdonald JG, et al (2015). Impact of tamoxifen on adipocyte lineage tracing : Inducer of adipogenesis and prolonged nuclear translocation of Cre recombinase. Mol Metab.

[B20] Cong L, Zhang F (2015). Genome engineering using CRISPR-Cas9 system. Methods Mol Biol.

[B21] Mou H, Kennedy Z, Anderson DG, Yin H, Xue W (2015). Precision cancer mouse models through genome editing with CRISPR-Cas9. Genome Med.

[B22] Zhang Ch, Zhou Y, Xie Sh, Yin Q, Tang Ch, Ni Z, et al (2018). CRISPR/Cas9-mediated genome editing reveals the synergistic effects of β-defensin family members on sperm maturation in rat epididymis. FASEB J.

[B23] Quadros RM, Miura H, Harms DW, Akatsuka H, Sato T, Aida T (2017). Easi-CRISPR: A robust method for one-step generation of mice carrying conditional and insertion alleles using long ssDNA donors and CRISPR ribonucleoproteins. Genome Biol.

[B24] Sipila P, Bjorkgren I

[B25] Zhou YS, Webb Sh, Lettice L, Tardif S, Kilanowski F, Tyrrell C, et al (2013). Partial deletion of chromosome 8 b-defensin cluster confers sperm dysfunction and infertility in male mice. PLOS Genet.

[B26] Sharan ShK, Pyle A, Coppola V, Babus J, Swaminathan S, Benedict J, et al (2004). BRCA2 deficiency in mice leads to meiotic impairment and infertility. Development.

[B27] Carvajal G, Brukman NG, Weigel Muñoz M, Battistone MA, Guazzone VA, Ikawa M, et al (2018). Impaired male fertility and abnormal epididymal epithelium differentiation in mice lacking CRISP1 and CRISP4. Sci Rep.

[B28] Carlson AE, Burnett LA, del Camino D, Quill TA, Hille B, Chong JA, et al (2009). Pharmacological targeting of native CatSper channels reveals a required role in maintenance of sperm hyperactivation. PLoS One.

[B29] Hsia K-H, Millar MR, King S, Selfridge J, Redhead NJ, Melton DW, et al (2003). DNA repair gene Ercc1 is essential for normal spermatogenesis and oogenesis and for functional integrity of germ cell DNA in the mouse. Development.

[B30] Sun F, Palmer K, Handel MA (2010). Mutation of Eif4g3, encoding a eukaryotic translation initiation factor, causes male infertility and meiotic arrest of mouse spermatocytes. Development.

[B31] O’Rand MG, Hamil KG, Adevai T, Zelinski M (2018). Inhibition of sperm motility in male macaques with EP055, a potential non-hormonal male contraceptive. PLoS One.

[B32] Imai H, Hakkaku N, Iwamoto R, Suzuki J, Suzuki T, Tajima Y, et al (2009). Depletion of selenoprotein GPx4 in spermatocytes causes male infertility in mice. J Biol Chem.

[B33] Crackower MA, Kolas NK, Noguchi J, Sarao R, Kaneko H, Kobayashi E, et al (2003). Essential role of Fkbp6 in male fertility and homologous chromosome pairing in meiosis. Science.

[B34] Fujihara Y, Lu Y, Noda T, Oji A, Larasati T, Kojima-Kita K, et al (2020). Spermatozoa lacking fertilization influencing membrane protein (FIMP) fail to fuse with oocytes in mice. Proc Natl Acad Sci U S A.

[B35] Martianov I, Brancorsini S, Catena R, Gansmuller A, Kotaja N, Parvinen M, et al (2005). Polar nuclear localization of H1T2, a histone H1 variant, required for spermatid elongation and DNA condensation during spermiogenesis. Proc Natl Acad Sci U S A.

[B36] Crapster JA, Rack PG, Hellmann ZJ, Le AD, Adams ChM, Leib RD, et al (2020). HIPK4 is essential for murine spermiogenesis. Elife.

[B37] Aydin H, Sultana A, Li Sh, Thavalingam A, Lee JE (2016). Molecular architecture of the human sperm IZUMO1 and egg JUNO fertilization complex. Nature.

[B38] Kawa S, Ito Ch, Toyama Y, Maekawa M, Tezuka T, Nakamura T, et al (2006). Azoospermia in mice with targeted disruption of the Brek/Lmtk2 (brain-enriched kinase/lemur tyrosine kinase 2) gene. Proc Natl Acad Sci U S A.

[B39] Yang J, Medvedev S, Yu J, Tang LC, Agno JE, Matzuk MM, et al (2005). Absence of the DNA-/RNA-binding protein MSY2 results in male and female infertility. Proc Natl Acad Sci U S A.

[B40] Tsuda M, Sasaoka Y, Kiso M, Abe K, Haraguchi S, Kobayashi S, et al (2003). Conserved role of nanos proteins in germ cell development. Science.

[B41] Nayernia K, Vauti F, Meinhardt A, Cadenas C, Schweyer S, Meyer BI, et al (2003). Inactivation of a testis-specific Lis1 transcript in mice prevents spermatid differentiation and causes male infertility. J Biol Chem.

[B42] Xu H, Beasley MD, Warren WD, Van Der Horst GTJ, Mckay MJ (2005). Absence of mouse REC8 cohesin promotes synapsis of sister chromatids in meiosis. Dev Cell.

[B43] Zeng X-H, Navarro B, Xia X-M, Clapham DE, Lingle ChJ (2013). Simultaneous knockout of Slo3 and CatSper1 abolishes all alkalization- and voltage-activated current in mouse spermatozoa. J Gen Physiol.

[B44] Kherraf Z-E, Christou‐Kent M, Karaouzene T, Amiri‐Yekta A, Martinez G, Vargas AS, et al (2017). SPINK 2 deficiency causes infertility by inducing sperm defects in heterozygotes and azoospermia in homozygotes. EMBO Mol Med.

[B45] Noda T, Lu Y, Fujihara Y, Oura S, Koyano T, Kobayashi S, et al (2020). Sperm proteins SOF1, TMEM95, and SPACA6 are required for sperm-oocyte fusion in mice. Proc Natl Acad Sci U S A.

[B46] Tanaka H, Iguchi N, Toyama Y, Kitamura K, Takahashi T, Kaseda K, et al (2004). Mice deficient in the axonemal protein Tektin-t exhibit male infertility and immotile-cilium syndrome due to impaired inner arm dynein function. Mol Cell Biol.

[B47] Xu B, Hao Zh, Jha KN, Zhang Zh, Urekar C, Digilio L, et al (2008). Targeted deletion of Tssk1 and 2 causes male infertility due to haploinsuf fi ciency. Dev Biol.

[B48] Noda T, Sakurai N, Nozawa K, Kobayashi S, Devlin DJ, Matzuk MM, et al (2019). Nine genes abundantly expressed in the epididymis are not essential for male fecundity in mice. Andrology.

[B49] Chung SSW, Wang X, Wolgemuth DJ (2016). Prolonged oral administration of a pan-retinoic acid receptor antagonist inhibits spermatogenesis in mice with a rapid recovery and changes in the expression of influx and efflux transporters. Endocrinology.

[B50] Busada JT, Geyer CB (2016). The role of retinoic acid (RA) in spermatogonial differentiation. Biol Reprod.

[B51] Chen Y, Zhu J-Y, Hong KH, Mikles DC, Georg GI, Goldstein AS, et al (2018). Structural basis of ALDH1A2 inhibition by irreversible and reversible small molecule inhibitors. ACS Chem Biol.

[B52] Al Noman MDA, Kyzer JL, Chung SSW, Wolgemuth DJ, Georg GI (2020). Retinoic acid receptor antagonists for male contraception: Current status. Biol Reprod.

[B53] Hawkinson JE, Sinville R, Mudaliar D, Shetty J, Ward T, Herr JC, et al (2017). Potent pyrimidine and pyrrolopyrimidine inhibitors of testis-specific serine/threonine kinase 2 (TSSK2). Chem Med Chem.

[B54] Srivastav A, Changkija B, Sharan K, Nagar GK (2018). Influence of antifertility agents dutasteride and nifedipine on Catsper gene level in epididymis during seperm maturation in BALB/c mice. Reproduction.

[B55] Matzuk MM, McKeown MR, Filippakopoulos P, Li Q, Ma L, Agno JE, et al (2012). Small-molecule inhibition of BRDT for male contraception. Cell.

[B56] Ayoub AM, Hawk LML, Herzig RJ, Jiang J, Wisniewsk AJ, Gee CT, et al (2017). BET bromodomain inhibitors with one-step synthesis discovered from virtual screen. J Med Chem.

[B57] Chávez JC, Ferreira JJ, Butler A, De La Vega Beltrán JL, Treviño CL, Darszon A, et al (2014). SLO3 K+ channels control calcium entry through CATSPER channels in sperm. J Biol Chem.

[B58] Rennhack A, Schiffer C, Brenker C, Fridman D, Nitao ET, Cheng YM, et al (2018). A novel cross-species inhibitor to study the function of CatSper Ca2+ channels in sperm. Br J Pharmacol.

[B59] O’Rand M G, Widgren EE, Sivashanmugam P, Richardson RT, Hall SH, French FS, et al (2004). Reversible immunocontraception in male monkeys immunized with eppin. Science.

[B60] O’Rand M, Silva EJR, Hamil KG (2017). Non-hormonal male contraception: A review and development of an Eppin based contraceptive. Pharmacol Ther.

[B61] D’Francisco F, Merlo M, Vercellini R, Blanco P, Barbeito C, Gobello C (2019). Effect of the indenopyridine RTI-4587-073 (l) on feline testicle. Anim Reprod Sci.

[B62] Tash JS, Attardi B, Hild SA, Chakrasali R, Jakkaraj SR, Georg GI (2008). A novel potent indazole carboxylic acid derivative blocks spermatogenesis and is contraceptive in rats after a single oral dose. Biol Reprod.

[B63] Mok K-W, Mruk D, Lie PPY, Lui W-Y, Cheng CY (2011). Adjudin, a potential male contraceptive, exerts its effects locally in the seminiferous epithelium of mammalian testes. Reproduction.

[B64] Kwon W, Park Y, Kim Y, You Y, Kim IC, Pang M (2013). Vasopressin effectively suppresses male fertility. PLoS One.

[B65] Lee JS, Kwon WS, Rahman MS, Yoon SJ, Park YJ, Pang MG (2015). Actin-related protein 2/3 complex-based actin polymerization is critical for male fertility. Andrology.

[B66] Peralta-Aris RD, Vivenes CY, Camejo MI, Pinero S, Proverbio T, Martinez E, et al (2015). ATPases, ion exchangers and human sperm motility. Reproduction.

[B67] Sanbe A, Tanaka Y, Fujiwara Y, Tsumura H, Yamauchi J, Cotecchia S, et al (2007). Alpha1-adrenoceptors are required for normal male sexual function. Br J Pharmacol.

[B68] Borges CS, Missassi G, Pacini ESA, Kiguti LRA, Sanabria M, Pupo S, et al (2013). Slimmer or Fertile? Pharmacological mechanisms involved in reduced sperm quality and fertility in rats exposed to the anorexigen sibutramine. Plos Med.

[B69] Zhang H, Yu H, Wang X, Zheng W, Yang B, Pi J, et al (2012). (S)-α-chlorohydrin inhibits protein tyrosine phosphorylation through blocking cyclic AMP-protein kinase a pathway in spermatozoa. PLoS One.

[B70] Balbach M, Fushimi M, Huggins DJ, Steegborn C, Meinke PT, Levin LR, et al (2020). Optimization of lead compounds into on-demand, nonhormonal contraceptives: Leveraging a public-private drug discovery institute collaboration. Biol Reprod.

[B71] Akbari A, Pipitone GB, Anvar Z, Jaafarinia M, Ferrari M, Carrera P, et al (2019). ADCY10 frameshift variant leading to severe recessive asthenozoospermia and segregating with absorptive hypercalciuria. Hum Reprod.

[B72] Shettya J, Sinvilleb R, Shumilinc IA, Minorc W, Zhanga J, Hawkinson JE, et al (2016). Recombinant production of enzymatically active male contraceptive drug target hTSSK2-localization of the TSKS domain phosphorylated by TSSK2. Protein Expr Purif.

[B73] Zhang H, Su D, Yang Y, Zhang W, Liu Y, Bai G, et al (2010). Some single-nucleotide polymorphisms of the TSSK2 gene may be associated with human spermatogenesis impairment. J Androl.

[B74] Salicioni AM, Gervasi MG, Sosnik J, Tourzani DA, Nayyab S, Caraballo DA, et al (2020). Testis-specific serine kinase protein family in male fertility and as targets for non-hormonal male contraception. Biol Reprod.

[B75] Conlon RA, Dam PPDD, Van D Animal models of dementia neuromethods.

